# A dual-layer quality assurance approach leveraging dose prediction for efficient review of automated contours of organs at risk in the brain in radiotherapy

**DOI:** 10.1016/j.phro.2025.100888

**Published:** 2025-12-06

**Authors:** Robert Poel, Amith Kamath, Ekin Ermiş, Jonas Willmann, Elias Rüfenacht, Nicolaus Andratschke, Peter Manser, Daniel M. Aebersold, Mauricio Reyes

**Affiliations:** a Department of Radiation Oncology, Inselspital, Bern University Hospital, and University of Bern, Bern, Switzerland; b ARTORG Center for Biomedical Research, University of Bern, Bern, Switzerland; c Department of Radiation Oncology, University Hospital Zurich, University of Zurich, Switzerland; d Department of Medical Physics, Memorial Sloan Kettering Cancer Center, New York, NY, USA; e Division of Medical Radiation Physics, Bern University Hospital, and University of Bern, Bern, Switzerland

**Keywords:** Autosegmentation, Quality assurance, Dose prediction, OARs, Brain

## Abstract

**Background and Purpose:**

Despite widespread adaptation of automatic segmentation (AS), manual review and adjustment of generated contours are still essential. This process is time-consuming and identifying clinically relevant corrections remains challenging. Inter-observer variability and the risk of overlooking significant errors further complicate the workflow. A dedicated quality assurance tool is highly relevant to assure quality and speed up the manual review task. The primary aim of this work is to identify critical segmentation errors while reducing unnecessary manual review, enabling efficient integration of AS into routine radiotherapy.

**Materials and Methods:**

We developed an evaluation assistant that assesses contour quality through the geometric measures Dice similarity coefficient and the Hausdorff distance. This was combined with a dose prediction model to determine the clinical relevance. The system was validated on 30 glioblastoma cases with ground truth and manually modified organ at risk (OAR) contours. A traffic light decision matrix classified contours based on geometric and dose parameters, flagging structures for human review.

**Results:**

Out of 507 analyzed OARs, 180 were classified as critical. Our approach identified 173 of these critical structures (sensitivity: 0.96, specificity: 0.55). The system flagged 317 organs (61%) as critical, effectively ruling out 39% as non-critical with only 7 false negatives comprising structures.

**Conclusions:**

Our dual-layer QA approach effectively identifies critical OAR segmentations with high sensitivity and acceptable specificity, potentially reducing manual review requirements significantly. By focusing on clinically relevant dose/volume metric endpoints, this method assures the quality of brain AS results in clinical radiotherapy practice.

## Highlights

• Novel “Swiss cheese” based quality assurance for auto-segmented contours in the brain.
• Independent layers give feedback on geometry and dose impact.
• Approach identifies 96% of critical organs, focusing on clinical relevance.
• Evaluation ensures quality of auto-segmentation results in clinical practice.

## Graphical abstract

The evaluation assistant detects treatment-critical segmentations. Auto-segmented input structures form the input for dose impact evaluator and a contour viability estimator based on an independent organ segmentation model. Dose, dose sensitivity and geometric parameters flag based on a “Swiss cheese” method and dose constraints any segmentations that need additional human evaluation.

**Figure:**
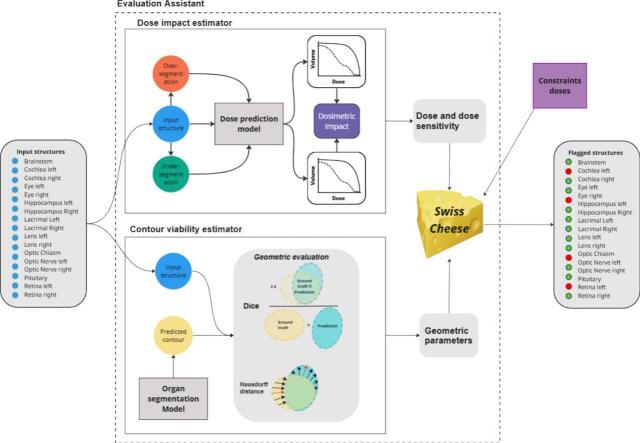

## 1 Introduction

In the radiotherapy workflow, accurately defining target volumes and organs at risk (OARs) is a critical step. Delineating the volume to be irradiated while sparing surrounding healthy tissues is essential for optimizing tumor control and minimizing toxicity. Manual contouring is time-consuming [1], [2] and exhibits high inter-observer variability depending on user experience, interpretation and image quality [3], [4]. Consequently, inter-observer variability remains a persistent issue in radiotherapy, with inconsistencies that may result in clinically significant errors [5], [6], especially in advanced high-precision radiotherapy modalities [7], [8], [9], [10], [11]. Notably, human errors in contouring are recognized as one of the major risks in radiotherapy according to the American Association of Physicists in Medicine [12].

The pursuit of automated segmentation (AS) as a solution is a major research focus [13], [14], [15], [16]. As outlined by Harrison et al. [16], auto-segmentation has progressed through four generations: from early intensity-based methods, to atlas-based approaches, machine learning methods, and recently, deep learning (DL). The evolution of these methods has led to significant improvements in accuracy, with DL models achieving quality comparable to manual contouring by experts [15]. The increasing performance and availability of commercial DL-based AS models have spurred their integration into clinical practice [16]. This is particularly true for OARs, where evaluations across anatomical regions suggest the problem is largely solved [17], [18], [19], [20]. However, AS remains prone to errors, as no DL model can achieve absolute accuracy. Consequently, expert validation remains essential, as mandated by clinical guidelines [21], [22].

The persistent need for human verification poses a challenge, as manual review is time-consuming and prone to overlooking errors [23]. The inherent uncertainty of DL predictions compounds this issue, as even state-of-the-art models may generate errors that are unpredictable and not obvious. This lack of reliability hampers clinical adoption and undermines confidence in automated solutions. Quality assurance (QA) methods that provide this confidence are therefore required. This was highlighted by Huyn et al., who emphasized that implementing QA tools is crucial for the clinical integration of artificial intelligence (AI) based models [24].

Recognizing this, recent work has emphasized QA’s critical role in enabling clinical use of AI-based segmentation [20]. While human oversight remains standard, AI is increasingly being explored as a tool for QA, with the potential to improve efficiency and performance [21], [25]. However, existing AI-based QA approaches have shown limitations in reliability and clinical relevance [26]. A reliable QA framework should not only detect geometric inaccuracies but also account for dose differences arising from the dose impact of contouring errors. Geometric evaluation alone can indicate varying degrees of contour deviation without establishing clinical significance. Because clinical impact in radiotherapy is ultimately reflected in tumor control and normal-tissue toxicity, endpoints that are directly correlated to the dose, and in particular, contour-induced dose differences provide an appropriate proxy for clinical relevance [20], [27], [28]. To address this gap, we propose a novel QA approach leveraging a multi-layered validation strategy inspired by the Swiss cheese model, a QA based system using different layers to catch errors [29], [30]. This builds on a growing body of work on automated dose-distribution prediction using artificial-intelligence methods, including deep-learning models that have demonstrated accurate 3D dose estimation across multiple treatment sites [31], [32], [33], [34], [35], [36], [37], [38] and extends these approaches to the quality-assurance context of contour-induced dose changes. In this model, each layer represents a distinct QA method, and the passage of errors becomes increasingly unlikely as additional layers are stacked. It is key however, that the layers are independent and complementary. We want to introduce dose as a layer because it is independent of the segmentation model, clinically relevant, and readily available through dose prediction models. As a second geometric validation layer, one can employ independently trained probability models, including methods proposed by Chen et al. [39], stochastic segmentation networks [40], or reverse classification accuracy techniques [41]. By combining geometric validation with dose prediction, our approach ensures that minor geometric errors that have no impact on the dose do not trigger unnecessary adjustments.

Therefore, the aim of this study is to develop and evaluate a dual-layer, dose-aware QA system (“evaluation assistant”) designed to detect clinically significant auto-segmentation errors while reducing unnecessary manual review. This work provides a first proof-of-concept evaluation for brain OAR segmentations.

## 2 Materials and methods

### 2.1 General outline

**Fig. 1:**
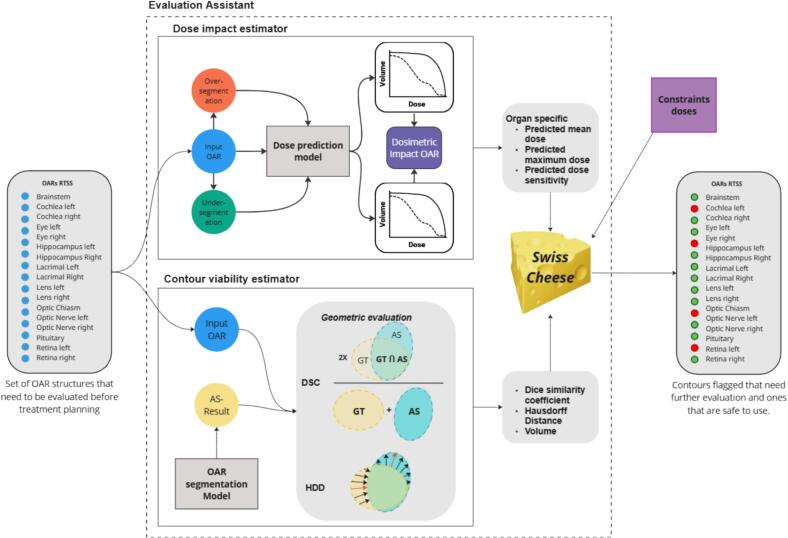
Evaluation assistant workflow for detecting treatment-critical segmentations. From left to right, auto-segmented OAR structure set (input to the critical-dose discriminator). The segmentations feed dose-impact and contour-variability estimators; their metrics go to an organ-specific discriminator that flags cases for additional human evaluation.

The evaluation assistant assesses contour acceptability through a combination of geometric validation and dose impact. Geometric validation compares segmentations to an independent AS model using Dice similarity coefficient (DSC) and Hausdorff distance (HDD) metrics. Dose/volume metric criticality is determined using a dose prediction model [35], defined by proximity to the clinical constraint doses or dose gradients. These assessments determine whether a segmentation requires review or is safe for clinical use (Fig. 1). Any non-flagged contour is safe to ignore, but flagged contours can have severe geometric errors, geometric inaccuracies that have potential impact on the dose, or have a dose close to the constraint dose. Since the individual models are not 100 % accurate, no specifications for the errors are given as this could have the potential to falsely bias the reviewers.

### 2.2 Data

For evaluation, we used a retrospective dataset, of 30 high-grade glioblastoma multiforme (GBM) patients, with signed consent and approved by the local ethics board, and treated with post-operative radiotherapy at Inselspital University Hospital, Bern. For each patient, four MR sequences (T1, T1c, T2, and FLAIR), planning CT, and ground truth contours for 17 OARs and target volumes were collected. Ground truth was established by consensus among three experienced radiation oncology professionals. These 30 cases were not used for training any AI models.

### 2.3 AI models

For the geometric validation layer, we used an in-house developed model based on a 2D U-net [42] to segment 17 OARs as defined by ESTRO-ACROP guidelines [43]. The model has been trained on multimodal MR images from 100 GBM and brain metastasis cases (50/50) using a 5-fold cross-validation (70 training, 10 validation, 20 testing) with ensembling, to ensure robustness. Clinical validation of this model has scored an average DSC of 0.78, with 88 % of the contours deemed acceptable after minor changes [44].

As dose predictor, we used a cascaded 3D U-Net architecture trained on 90 carefully curated GBM plans (which includes the 50 cases for the segmentation model). The details of this model can be found in the supplementary materials. All plans used volumetric modulated arc therapy (VMAT) with double full co-planar arcs and were prescribed 60 Gy in 30 fractions, following clinical constraints and OAR prioritization. Training inputs included normalized CT scans, 3D dose volumes, and binary masks of targets and OARs, with the model predicting continuous-valued 3D dose distributions. The model has shown a dose score of 0.94 Gy and a mean DVH score of 1.94 Gy [45]. In terms of sensitivity to segmentation variability, the model demonstrated a high correlation between predicted doses and calculated doses for 10 contour variations [46]. More detailed information on the models can be found in the supplementary materials.

### 2.4 Clinical dose/volume evaluation – Reference validation

**Fig. 2:**
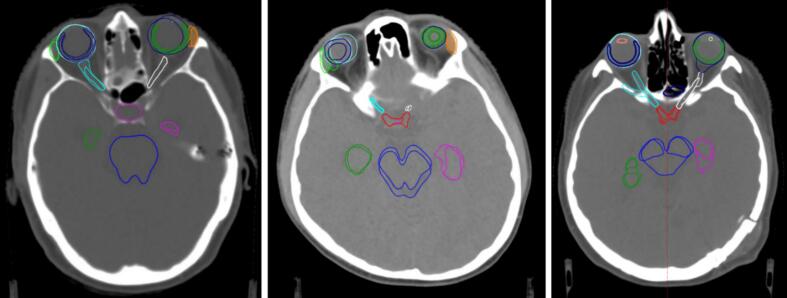
Examples of the manual manipulations of the ground truth organs for the test set. This shows the nature and extent of the made adjustments.

To establish a reference standard, we used 30 GBM cases with ground truth contours for all 17 OARs and the planning target volume (PTV). We generated a test set with realistic contouring errors by manually modifying the ground truth contours using the standard tools in the Eclipse treatment planning system (Varian Medical Systems, Palo Alto, CA) (Fig. 2). For each case, an optimal reference plan was created from ground truth contours following a strict planning protocol. Alternative plans were generated by substituting modified contours into copies of the reference plan, followed by re-optimization and dose recalculation, as described in [27] and illustrated in Fig. 3 of the supplementary materials.

**Fig. 3:**
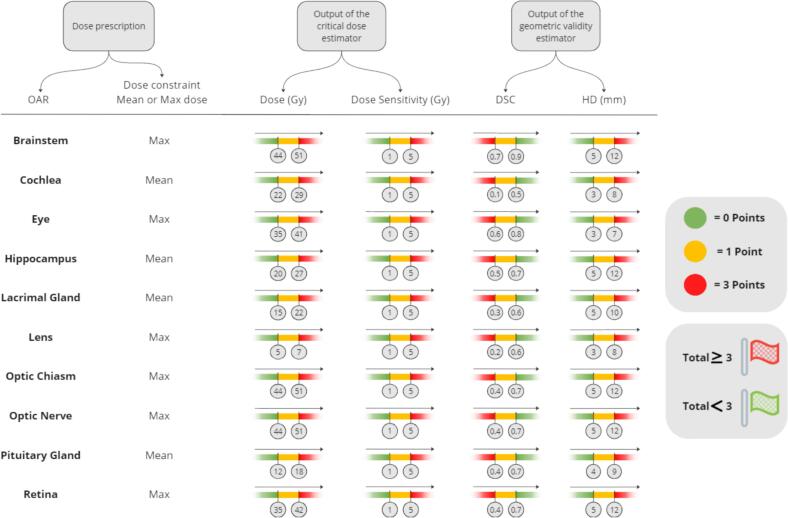
*The proposed* traffic-light decision matrix used by the evaluation assistant to score dose/volume and geometric parameters. For each OAR, the mean or maximum dose is selected per its constraint. Structures are scored by color (green = 0, yellow = 1, red = 3); totals ≥ 3 are flagged for further review. (For interpretation of the references to color in this figure legend, the reader is referred to the web version of this article.)

For each OAR, the maximum and minimum dose differences between the two plans were determined. We performed a geometric assessment of the ground truth and manually adjusted contours using DSC and HDD metrics. Based on these metrics and constraint doses, we evaluated the clinical acceptability of each manually adjusted contour with a traffic light system (Fig. 3). For each organ, the mean or maximum dose was selected according to OAR-specific dose constraints. Each structure received points based on the color assigned by the traffic light result. Colors were defined by organ- and metric-specific thresholds: red indicates a critical metric requiring evaluation; green indicates acceptability and no clinical concern. Green equals zero points, yellow one point, and red three points. A total of three or more points across the four metrics flags the structure for further review.

### 2.5 Evaluation assistant

The geometric validity estimator assessed segmentation accuracy by comparing it with predictions from the independent AS model using DSC and 95 % HDD metrics. Evaluation segmentations and AS results were registered to the T1c MR sequence frame of reference, with assessment performed in the discrete domain.

**Fig. 4:**
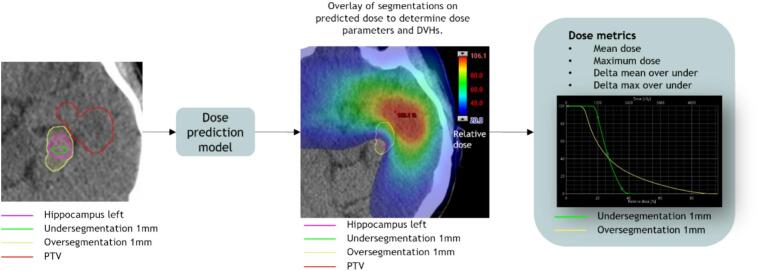
Schematic overview showing how the dose and dose sensitivity are determined on the evaluation segmentation. Based on the segmentation result an over and under segmentation is determined. Both scenarios are overlayed on the predicted dose, which will provide two dose parameters (mean dose and max dose) for the structure as well as different dose volume histogram (DVH) curves.

For the critical dose estimator, the dose prediction model generated optimal 3D dose distributions using the evaluation segmentation, planning CT, and PTV as inputs. From these predictions, dose-volume histograms (DVH) and dose parameters for each OAR were calculated. Dilated and eroded versions of evaluation segmentations were created to simulate over- and under-segmentation scenarios and determine the dose sensitivity of each contour (Fig. 4). The dilation/erosion extent was organ-specific and based on the mean 95 % HDD derived from clinical evaluation and mean organ volume. The specific numbers can be found in Table 1 of the supplementary material. Generally, erosion was less aggressive, to prevent unrealistic results in smaller structures. Mean and maximum doses to the over- and under-segmented structures were determined to calculate dose differences. The critical dose estimator outputs predicted mean and maximum doses for each contour, and absolute delta mean and maximum doses of over-/under-segmentations. The delta mean and delta maximum doses served as dose sensitivity measures directly related to clinical constraint doses.

### 2.6 Assessing the evaluation assistant

**Fig. 5:**
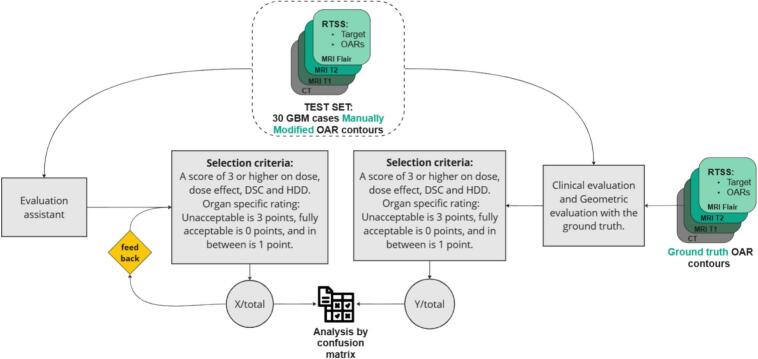
Definition of the critical dose/volume metric events for both the clinical and dose/volume evaluation used as the reference and for the critical dose estimator that we want to validate. A critical event is defined as either one or both conditions being met. Because the critical dose estimator requires a high sensitivity, additional safety margins are defined to reduce the chances of having false negatives. The critical events as a fraction of the total contours are compared for both methods in a confusion matrix.

Using inputs from both estimators, the evaluation assistant applied a decision scheme to flag contours requiring further review. Our analysis included 17 organs across 30 cases (507 segmentations in total). Results were compared to reference data using a confusion matrix to determine true/false positives and negatives, enabling the calculation of sensitivity and specificity. Selection criteria were adjustable based on the confusion matrix results (Fig. 5).

## 3 Results

Of the 507 OARs analyzed, 180 were classified as critical by the clinical evaluation, according to the criteria in Fig. 3. Using our evaluation assistant approach, we identified 173 of these critical structures, yielding a sensitivity of 0.96 and a specificity of 0.55. This approach flagged 317 organs (61 % of all OARs) as critical, ruling out 39 % of OARs as non-critical, with only 7 false negatives. False negatives occurred in 6 different cases and comprised one optic nerve, one eye, one lens, one retina, two pituitary glands, and one cochlea. An overview of the false positives and their metric values is provided in Table 1.

**Table 1:** Overview of contours defined as false negatives by the evaluation assistant. The metric values of the clinical evaluation (reference) and the evaluation assistant, including the traffic light colors are provided.

## 4 Discussion

This study demonstrates the utility of a dose prediction model identifying dose critical OARs in AS results. Our critical dose estimator successfully identified 93.8 % of dose critical AS OARs, flagging 45 % of cases as critical while classifying the remaining 55 % as less critical, reducing the need for manual review.

Quality assurance has evolved from machine learning and statistical models [47], [48], [49], [50], [51], [52], [53], [54] to advanced DL techniques. Traditional methods face inherent limitations in providing certainty; recent advancements remain promising but limited. Uncertainty estimation methods, which leverage probability metrics from segmentation models to train secondary classifiers [39], have shown that model-derived uncertainty does not consistently predict segmentation accuracy and may introduce bias. Similarly, out-of-distribution detection approaches [55], [56], while identifying deviant input samples, often lack sensitivity and miss incorrect segmentations when trained on synthetic or unrelated data.

Several notable advances have emerged: Isaksson et al., developed a model that effectively predicted DSC metrics [25]. Altman et al.'s knowledge-based geometrical approach detected 40 of 42 errors despite producing 9 false positives [48], Hui et al.'s parametric statistical approach identified 37 % of minor errors, 85 % of major errors, and 94 % of manually induced errors [51], and Chen et al.'s ResNet-101 based QA network achieved 90 % accuracy [57]. However, these approaches generally lack comprehensive clinical accuracy validation, leaving failure modes and practical limits uncertain; furthermore, ground-truth error definitions vary (metric-based, expert evaluation, or individual assessment). In this study, we complement a geometric check with a dose-aware layer and verify contours deemed critical through dose evaluation. Our “Swiss cheese” framework is modular: uncertainty estimation and out of distribution detection can be incorporated as additional, independent layer, or substitute the second segmentation layer. Provided they are calibrated, reliable, and complementary, additional layers have can to improve the evaluation tool.

Our critical dose estimator operates independently of the AS models and targets clinically relevant dose/volume metric endpoints, complementing geometric validation. The tool is designed to assist, not replace clinician review, by triaging where attention is most needed: contours are flagged when either an independent geometric check or a dose-aware assessment against organ-specific constraints suggests potential clinical impact, while non-flagged cases have passed both checks. This dose awareness addresses the known difficulty of inferring clinical consequence from geometry alone and helps distinguish benign deviations from those likely to matter. Although a combined geometric/dose approach has the potential to reduce review burden and standardize decisions, our study is retrospective, prospective evaluation is needed to demonstrate workflow effects and confirm sustained trust in clinical radiotherapy practice.

An effective QA system requires high sensitivity and sufficient specificity for meaningful reduction in manual review. While our critical dose estimator demonstrated promising sensitivity, some critical cases were missed due to limited accuracy to predict doses for specific OARs. Three false negatives involved cochlear structures, where the dose predictor showed inaccuracy in estimating cranio-caudal dose fall-off, a limitation attributable to its 2D axial slice-based training. The dose prediction model scored comparable to the highest ranking results of the OpenKBP [58], with a dose score to the brain, of less than 1 Gy. However, comparison is difficult since the OpenKBP challenge was performed in head and neck cases, and dose score metrics cannot be directly compared between anatomies. Similar studies on dose prediction in the brain, were difficult to compare. Irannejad et al., with a prediction model for IMRT in gliomas, provided whole body results in mean square error without specifying units [59]. Miao et al., showed results of dose prediction models for the Cyberknife and reported accuracy by gamma passing rates [60]. Nevertheless, we anticipate improved performance with 3D training approaches and by combining the critical dose estimator with complementary geometric validation, to be explored in future research.

Several limitations warrant consideration. This feasibility study tested only brain OARs in glioblastoma patients treated with VMAT, the treatment protocol used to train our dose prediction model. The model’s inability to account for overlapping structures prevented accurate dose prediction for the lens and retina. Our evaluation also included only 30 cases from a single institution (507 AS results) due to the need to train multiple deep learning models without case overlap among the AS, dose prediction, and evaluation datasets.

The implemented traffic light system is based on thresholds that may appear arbitrary, although it demonstrated good performance with higher sensitivity than a random forest classification model. This sensitivity aligns with the system’s primary goal of improving QA in the dose prediction workflow. The numerical thresholds were selected from experience in geometric similarity studies and the clinical relevance of constraint doses, though alternative configurations could be used. The framework offers clear advantages in comprehensive assessment, enabling simultaneous evaluation of multiple model aspects. The system can independently assess predicted dose distributions or geometric validity and combine results while accounting for organ-specific dose constraints. This multifaceted approach yields interpretable results for clinical implementation. The chosen values have true clinical meaning and are therefore explainable. However, there is no consensus—apart from dose constraints—on acceptable dose increases or decreases. It will be important for the community to discuss this issue and work toward generalizable normal tissue complication probability models. The flexible framework also allows penalization of individual components or combined metrics, providing an evaluation that captures the complex requirements of radiotherapy plan assessment.

Despite these limitations, this dose prediction-based QA approach could be adapted for various radiotherapy treatments. AS models are widely available for various anatomical locations, and numerous independent segmentation models can provide probability maps for different regions [61], [62], [63], [64], [65]. The optimal geometric validation model to pair with dose prediction remains an open question. Stochastic segmentation networks based on human contour variance represent one promising option, potentially reducing correlated errors in a “Swiss cheese model” of failure prevention [40]. Furthermore, dose-sensitivity estimation should become more data-driven. In this version, isotropic dilation/erosion per organ, based on the HDD and mean volume, was chosen for transparency and reproducibility in the absence of patient-specific uncertainty information. The approach is conservative and may understate direction-specific errors, so we gate decisions by dose thresholds relative to OAR constraints. Future work will replace isotropy with anisotropic, data-informed contour variability derived from real-world correction data or uncertainty maps from segmentation models. [66].

The evaluation assistant depends critically on accurate dose prediction models. However, current models are trained on data specific to individual diseases, protocols, and institutions, making them non-generalizable across different treatment settings. Since generalizable models do not exist, implementing accurate dose prediction requires training numerous separate models for each disease, institution, and treatment modality creating substantial demands for model development and training data.

Generalizable prediction models would therefore be extremely valuable, with initiatives like the generalizable dose prediction challenge (GDM-HMM), representing promising steps in this direction [67]. Additionally, our method assumes target structures are available for accurate dose prediction; when both organs-at-risk and targets are auto-segmented simultaneously, physicians must first verify targets before applying the QA model.

Nevertheless, the dose predictor remains the key element in achieving radiotherapy-relevant QA. It provides treatment quality-related information complementary to geometric similarity metrics. To our knowledge, this represents the first attempt to utilize a dose prediction model for auto-segmented OAR quality assurance.

In conclusion, our dose prediction model effectively identifies dose critical OAR segmentations, demonstrating high sensitivity and good specificity when validated against clinical quality plans based on both auto-segmented and ground truth contours. Although more work is required to improve model accuracy in overlapping organs and cranio-caudal dose fall-off, these results offer promising perspectives for a comprehensive QA system integrating geometric validation with dose awareness for efficient evaluation of brain AS results.

## Declaration of generative AI and AI-assisted technologies in the writing process

During the preparation of this work the author(s) used Grammarly in order to improve grammar and writing style. After using this tool/service, the author(s) reviewed and edited the content as needed and take(s) full responsibility for the content of the publication.

## CRediT authorship contribution statement

**Robert Poel:** Conceptualization, Methodology, Validation, Formal analysis, Investigation, Writing – original draft. **Amith Kamath:** Writing – review & editing. **Ekin Ermiş:** Data curation. **Jonas Willmann:** Data curation. **Elias Rüfenacht:** . **Nicolaus Andratschke:** Conceptualization, Methodology, Validation. **Peter Manser:** Conceptualization, Methodology, Validation. **Daniel M. Aebersold:** Conceptualization. **Mauricio Reyes:** Conceptualization, Methodology, Validation, Writing – original draft, Supervision.

## Declaration of competing interest

The authors declare that they have no known competing financial interests or personal relationships that could have appeared to influence the work reported in this paper.
