# Supplementary material for: A dual-layer quality assurance approach leveraging dose prediction for efficient review of automated contours of organs at risk in the brain in radiotherapy
Source: Phys Imaging Radiat Oncol. 2025 Dec 6;36:100888. doi: 10.1016/j.phro.2025.100888 (PMC12765091; doi:10.1016/j.phro.2025.100888)
Supplement: Supplementary Data 1 [file mmc1.pdf]

Supplementary Material

1. Materials and methods: extended

1.1 General outline

The evaluation assistant assesses contour quality through geometric validation and dosimetric impact. Geometric validation compares segmentations to an independent auto-segmentation (AS) model using Dice similarity coefficient (DSC) and Hausdorff distance (HD) metrics. Dosimetric criticality is determined using a dose prediction model [1] that evaluates the predicted dose and dose sensitivity, the potential dose change when adjusting the contour, for each organ. Combined with clinical constraint doses, these assessments determine whether a segmentation requires review or is safe for clinical use (Figure 1).

Since the single models for auto-segmentation and dose prediction can each have their own flaws, we opted to combine them in a Swiss cheese model. The Swiss cheese model, originally developed by psychologist James Reason, is a widely used framework in healthcare quality assurance [2,3]. The model conceptualizes safety systems as multiple layered barriers, each containing inherent weaknesses or "holes" that can shift due to factors such as human error, equipment failures, or system pressures. In this model, adverse events occur when holes in multiple safety barriers temporarily align, creating a pathway for errors to reach patients despite the presence of individual protective measures. The framework emphasizes that effective quality assurance requires multiple overlapping safety layers as no individual barrier is completely reliable.

This study validates whether the evaluation assistant can identify dosimetrically critical segmentations based on true dosimetric evaluation using 30 glioblastoma (GBM) cases with ground truth OAR contours and manually modified contours.

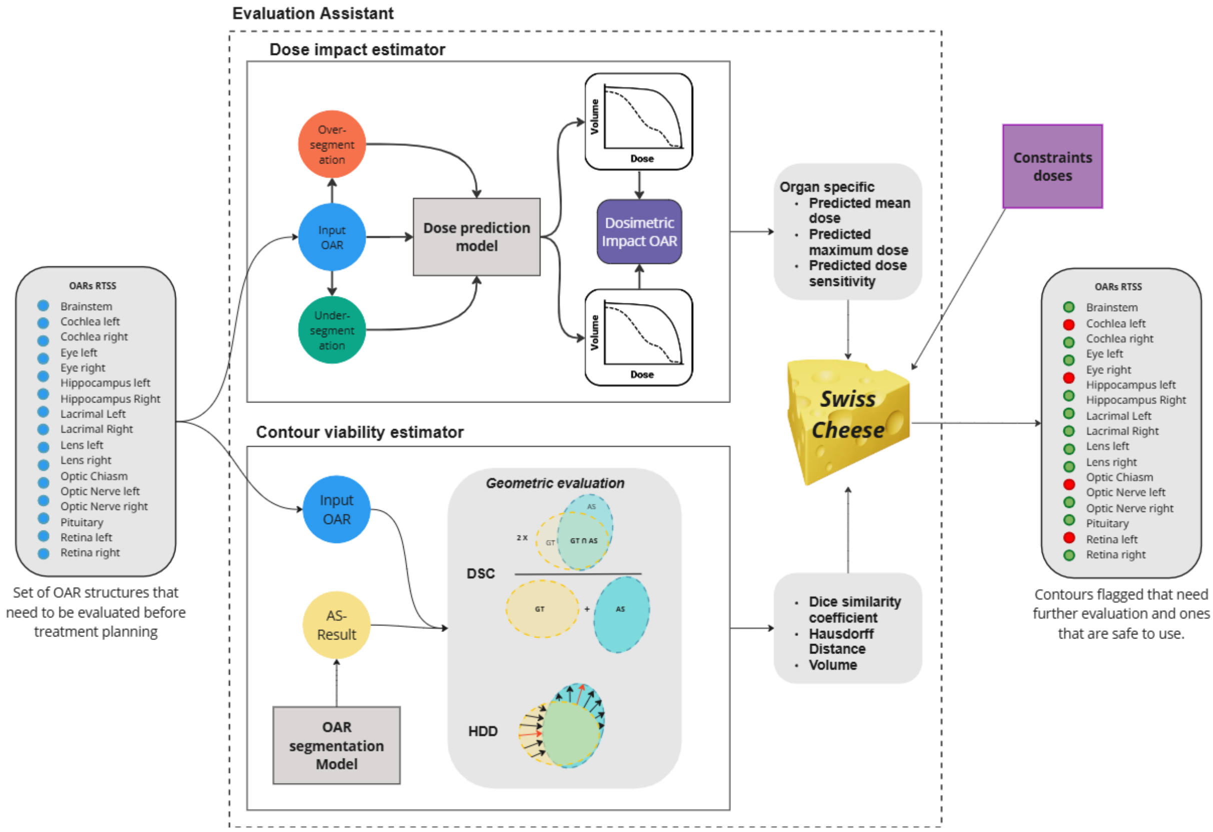

Figure 1: *Schematic overview of the evaluation assistant that enables the detection of segmentations that are*

critical for the treatment. On the left, the input for the critical dose discriminator is a structure set with auto-segmented OARs. The segmentations will be used as input for the dose impact estimator and the contour variability estimator. Both estimators provide input metrics for the organ specific discriminator to judge whether the segmentation needs to be flagged for additional human evaluation.

1.2 Data

To evaluate our proposed method, we used a retrospective dataset of 30 high grade GBM patients. The 30 cases were randomly selected from a larger cohort of 140 cases who have been treated with post-operative radiotherapy at Inselspital University Hospital Bern between 2016 and 2021. The cohort had an average age of 61.6 (± 12.4) years at treatment. For each patient, four MRI sequences (T1 weighted, T1 with contrast, T2 weighted, and FLAIR) and planning CT were collected, along with ground truth contours for 17 OARs and target volumes. The ground truth was established by mutual consensus among three experienced radiation oncology professionals by means of an iterative evaluation and adjustment process. The 30 cases for evaluation are not used for the training of any of the used AI models.

1.3 AI models

An in-house DL model was trained for auto-segmentation (AS) of intracranial organs at risk (OARs). The segmentation task involved annotating 17 distinct OARs based on Scoccianti et al. and the ESTRO-ACROP guidelines [4,5]. To manage and consider memory constraints and training dataset size, a 2D U-Net was employed [6]. The model was trained on four MR sequences (T1 with contrast, T1 without contrast, T2 and Flair) (CT was excluded as it did not provide improvements), following a similar pipeline as in Abayazeed et al. [7]. The methodological enhancements incorporated into the 2D U-Net included the addition of batch normalization and dropout. The cross-entropy loss function was minimized during training using an Adam optimizer. A learning rate schedule was adopted, and an early stopping mechanism was applied to prevent overfitting. Data balancing measures and diverse regularization techniques, such as affine transformations and Gaussian noise, were implemented. A robust five-fold cross-validation strategy was employed, utilizing a dataset of 70 subjects for training, 20 for testing, and 10 for validation.

For dose prediction model training, 95 cases were selected from 140 GBM cases. Three radiation oncology professionals curated planning target volume (PTV) and organs at risk (OARs). Cases were divided into training (60), validation (15), and test (20) sets. All cases were planned to receive 60 Gy in 30 fractions according to the ESTRO-EANO guidelines [8] using Eclipse treatment planning system (TPS) V15.06.05 (Varian Medical Systems, Palo Alto). OARs were subject to dose constraints according to a priority list (Table 1). Plans used volumetric arc technique (VMAT) with double full co-planar arc and 6 MV beams with flattening filter. Optimization used the photon optimizer with Anisotropic Analytical Algorithm [9]. After calculation, the dose was normalized so 50% of PTV was covered by 100% of prescribed dose, according to the institutional standard.

Planning CT and structures were converted from DICOM to NIfTI using the PyRaDise package [10]. RTSS files were divided into 14 separate 3D binary masks. Input files consisted of 16 3D volumes per case: planning CT, dose distribution, PTV binary mask, and 13 OAR binary masks (Figure 1).

We trained a two-level cascaded 3D (C3D) U-Net [11] as the dose prediction network, where the second U-Net input is the first U-Net output concatenated with the original input. The C3D model incorporates global and local anatomical features and showed best results in the OpenKBP challenge [12].

Model input was the normalized CT (Hounsfield units scaled between 0 and 1) volume and binary segmentation masks for 13 OARs and target volume. Output was continuous-valued 3D dose (upscaled from [0,1] to [0, 70 Gy]). Loss was computed as:

$$Loss = 0.5 * L1(reference, A) + L1\ (reference, B)$$

where *A* and *B* are outputs of first and second U-Nets, respectively. *Reference* indicates reference dose, and $L1$ refers to $L1$ loss. Volumes were resampled to 128^3 voxels due to GPU memory constraints. Hyperparameters were unchanged from original implementation [11], except the input binary masks were updated to 14. Weights were randomly initialized using "He" method [13]. Training ran for 80,000 iterations, saving the best validation model. Experiments used PyTorch1.12 on NVIDIA RTX A5000 GPU. The model was trained five times with different random seeds. Each training took 24 h. Single inference takes 1-2 min on standard a PC, and 15 s when using a GPU. A description of the evaluation of the model can be found in previous published work [14,15]

1.4 Clinical dosimetric evaluation – Reference validation

To establish a reference standard, we used the 30 selected GBM cases with ground truth contours for all 17 OARs and the planning target volume (PTV). While we did not have real world data of deviations at our disposal, we opted to create a test set based on manual modifications of the ground truth. This enabled us to provide a test set that includes realistic deviations with specific situations such as a total missing contour, left right mix-ups, translations, deformations, missing parts and random false positives, with a realistic deviation in terms of DSC ranging from 0 to 0.99 depending on the typical organ. The basis for this approach was defined on inter-rater variability data and by previous studies that looked at the impact of contour variations on the dose [16,17]. The manipulations were performed with the contouring tools available in the Eclipse treatment planning system (TPS). Contours have been translated and rotated in different directions, enlarged, shrunken, deformed and parts of the contours have been added or erased. Finally this resulted in a test set with an average deviation of 0.73 (SD: 0.24) and 0.6 mm (SD: 10.7) for the DSC and the HD respectively, with respect to the ground truth. A visual representation is given in figure 2.

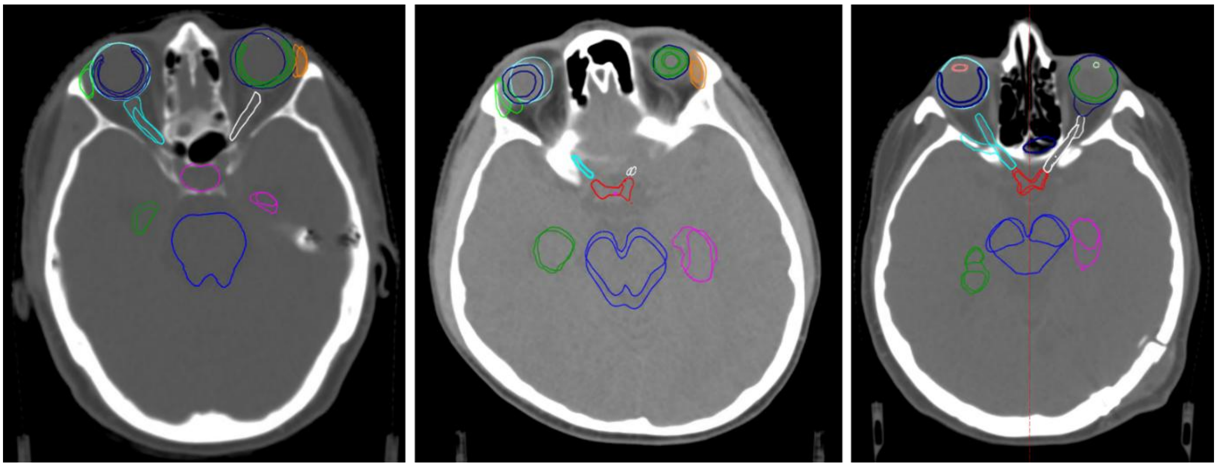

Figure 2: *Examples of the manual manipulations of the ground truth organs for the test set. This shows the the nature and extent of the made adjustments.*

For every case, a reference RT plan was generated based on the ground truth structures. The Clinical Target Volume (CTV) was defined as resection cavity and remaining enhancing tumro including a 15mm isotropic margin adjusted for the anatomy, per ESTRO-EANO guidelines [8]. A 3 mm margin was added to form the PTV. Prescription dose was 60 Gy in 30 × 2.0 Gy fractions. OARs and constraint doses are in Table 1. A co-planar VMAT plan with double full arc and 6 MV X-ray flattening filter free

beams was optimized using the anisotropic analytical algorithm. Plans were accepted when all constraints were met and normalized so 100% of prescribed dose covered 50% of PTV.

Table 1. Structures and dose prescription

	Dose prescription	
PTV - Reference only		60 Gy
Reference and alternative	Constraint dose	
Brainstem Surface	Max dose to 1%	≤ 60 Gy
Brainstem Center	Max dose to 1%	≤ 54 Gy
Eye (L+R)	Max dose to 1%	≤ 10 Gy
Chiasm	Max dose to 1%	≤ 55 Gy
Optic Nerve (L+R)	Max dose to 1%	≤ 55 Gy
Hippocampus (L+R)	Dose to 40% of volume	≤ 7.3 Gy
Lens (L+R)	Max dose to 1%	≤ 10 Gy
Lacrimal gland (L+R)	Mean dose	≤ 25 Gy
Cochlea (L+R)	Hard: Mean Dose	≤ 45 Gy
	Soft: Mean Dose	≤ 32 Gy
Retina (L+R)		≤ 45 Gy
Pituitary	Hard: Mean Dose	≤ 45 Gy
	Soft: Mean Dose	≤ 20 Gy

For alternative structure sets, new plans were created keeping all treatment parameters except OAR structures unchanged. Reference plans were duplicated and reference OARs substituted with alternative OARs. Beam orientation, prescription, constraints and optimization weights remained unchanged, then plans were re-optimized. This resulted in slightly different dose distributions due to different OAR orientations. These plans were also normalized so 100% of prescribed dose covered 50% of PTV.[16] The process is graphically explained in Figure 3.

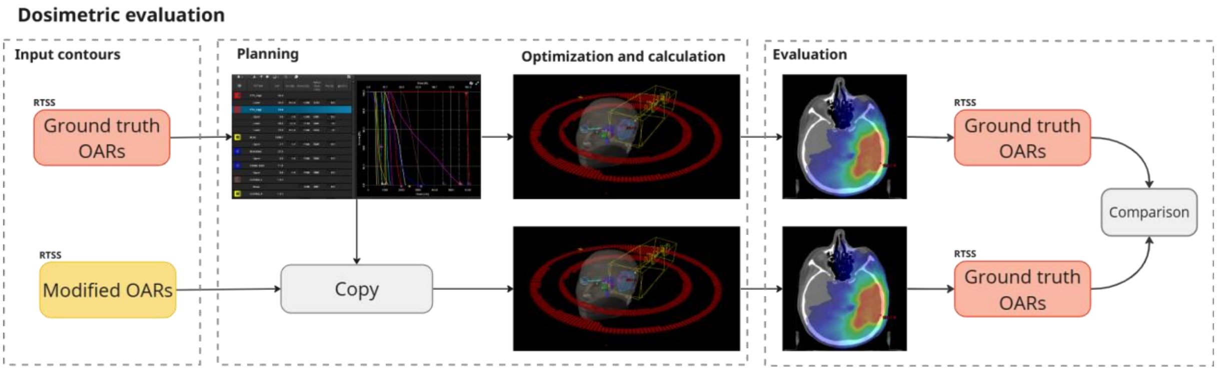

***Figure 3**: A graphical overview of the planning process for the clinical evaluation of the dose. A reference plan is defined on the ground truth organs at risk. A viable plan is constructed conform to clinical standard, the prescription dose for the PTV was set to 60 Gray (Gy) in a conventional scheme (30 × 2.0 Gy), by means of a two full co-planar arcs VMAT plan in the treatment planning system Eclipse (Varian Medical, Palo Alto, US). In a copy of the plan, the structure set is substituted for the on containing the modified OARs. While keeping all the optimization parameters the same, the optimization is re-run and the dose is calculated. The dose evaluation of both plans takes place on the ground truth OARs.*

A dosimetric evaluation quantified maximum and minimum dose differences for each OAR between the two plans. We also performed a geometric assessment of the ground truth and the manually adjusted contours using DSC and HD. Clinical acceptability was then judged with a traffic-light system (Figure 4) applied per organ and per metric—DSC, HD, absolute dose, and delta dose. For each OAR,

the mean or maximum dose was selected according to its clinical constraint. Color thresholds were defined as follows: green (safe, 0 points), orange (disputable, 1 point), and red (unacceptable, 3 points). Dose thresholds were anchored to organ-specific constraint doses; dose sensitivity was set such that <1 Gy was acceptable and >5 Gy unacceptable. Geometric thresholds (DSC, HD) were derived from historical inter-rater variability for each OAR.

The scoring scheme was designed so that any single unacceptable result (red) triggers a flag, whereas disputable results (orange) depend on corroboration from the other metrics. To operationalize this, we used a cumulative threshold of ≥3 points: an unacceptable finding in either dose or geometry immediately exceeds this threshold and is flagged; a single disputable finding in dose or geometry is not flagged; if both dose and geometry are disputable, the case is flagged. This ensures that segmentations with small geometric deviations are not flagged when the dose remains clearly safe, but are escalated when the dose is close to being clinically impactful. Each structure's colors were converted to points (green = 0, orange = 1, red = 3), and totals ≥3 prompted further evaluation. We have clarified these design choices in the revised manuscript.

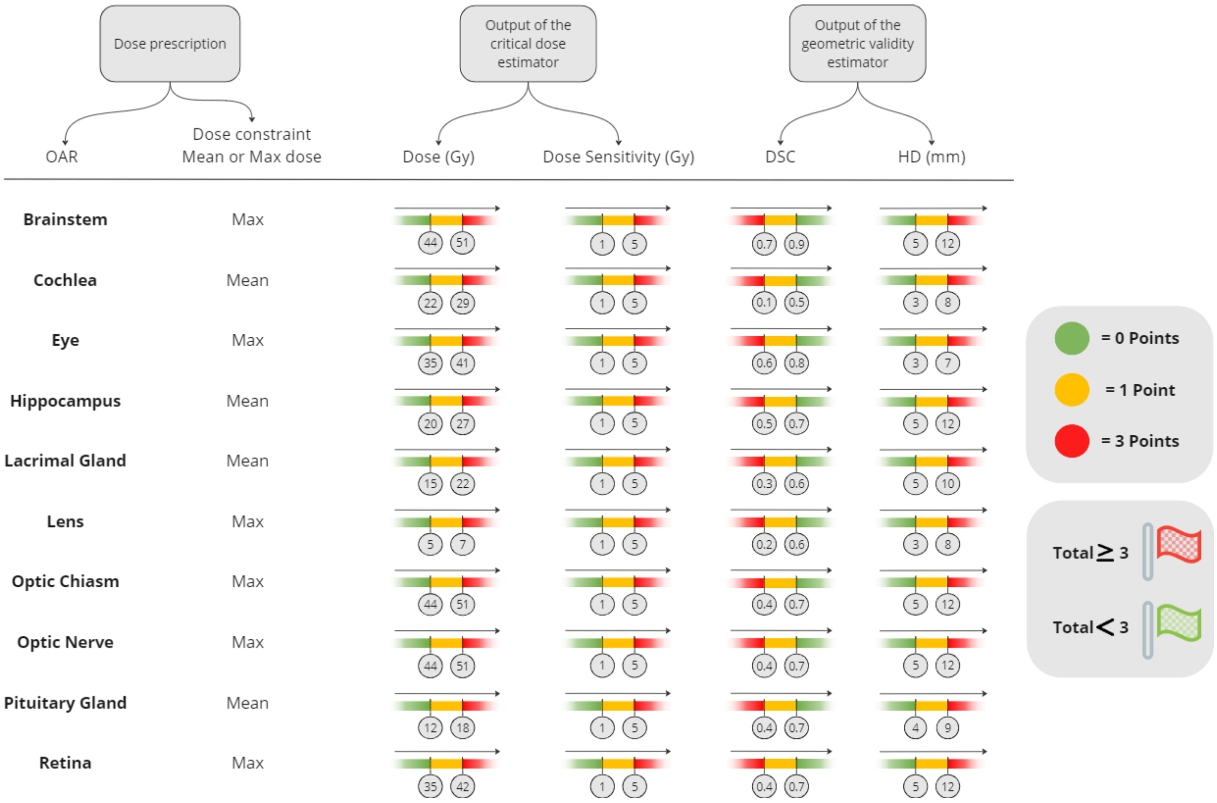

***Figure 4**: The proposed traffic light decision matrix of the evaluation assistant for scoring dosimetric and geometric parameters. For each organ, the mean or maximum dose is chosen based on OAR-specific dose constraints. Each structure receives points depending on the color received by the traffic light result. Green is zero points, yellow is one point and red is 3 points—a total of 3 points or higher means the structure will be flagged for further evaluation.*

2.5 Evaluation assistant

The geometric validity estimator validates segmentation geometric correctness by comparing it with predictions from our auto-segmentation model. Both the ground truth contours and the manually modified contours are exported from the Eclipse TPS in Dicom format. The results of the auto-segmentation model are also in Dicom format. The contours are all registered to the T1c MR sequence frame of reference with a grid size of 1x1x1 mm. The contours are converted 3D binary masks in NifTi format using the PyRaDiSe package [10]. The specific contours within each case, GT,

modified, and auto-segmented are geometrically analysed to define the DSC and the 95% HD metrics.

The dose prediction model generates optimal 3D dose distributions using evaluation segmentations, planning CT, and PTV as inputs. From these predictions, dose-volume histograms (DVH) and dose parameters for each OAR are calculated. Dilated and eroded versions of evaluation segmentations are also created to simulate over- and under-segmentation scenarios and act as a means to determine the dose sensitivity of a specific contour. The extent of dilation/erosion is specific to each organ based and is loosely based on the mean 95% HD distance derived from clinical evaluation and is than adjusted based on the mean organ volume (Table 2). Generally, erosion is less aggressive to prevent unrealistic results in smaller structures. Mean and maximum doses to the original, over-, and under-segmented structures are determined to calculate dose differences. The critical dose estimator outputs predicted mean and maximum doses for each evaluation segmentation, and absolute delta mean and maximum doses of over/under-segmentations.

Table 2*: Included OARs and their defined dilation and erosion values*

	Mean 95% HDD (mm)	Mean Volume (cm^3)	Dilation (mm)	Erosion (mm)
**Brainstem**	4.6	25.3	3	2
**Cochlea Left**	3.3	0.2	1	1
**Cochlea Right**	2.9	0.2	1	1
**Eye Left**	3.5	8.1	2	2
**Eye Right**	3.4	8.1	2	2
**Hippocampus Left**	4.0	2.0	2	2
**Hippocampus Right**	4.5	2.0	2	2
**Lacrimal Left**	4.0	0.7	2	1
**Lacrimal Right**	3.7	0.8	2	1
**OpticChiasm**	4.9	0.4	3	1
**OpticNerve Left**	6.8	1.2	3	1
**OpticNerve Right**	7.1	1.2	3	1
**Pituitary**	3.5	0.6	2	2

Besides the dose to the evaluation segmentation, the mean and maximum dose to the over- and under-segmentation are determined to calculate their dose difference. The delta mean dose and the delta max dose can be used as a dosimetric impact estimation measure that is directly related to the clinical constraint doses. A more general metric of the dosimetric impact estimation is provided by calculating the area between the curves (ABC) of the over and under-segmentation DVH curves, see Figure 4. This ABC measure will be a fraction of the total DVH area (Figure 4). The result of the critical dose estimator is a prediction of the mean and maximum dose for each evaluation segmentation, the absolute delta mean dose delta max dose of the over- and under-segmentation.

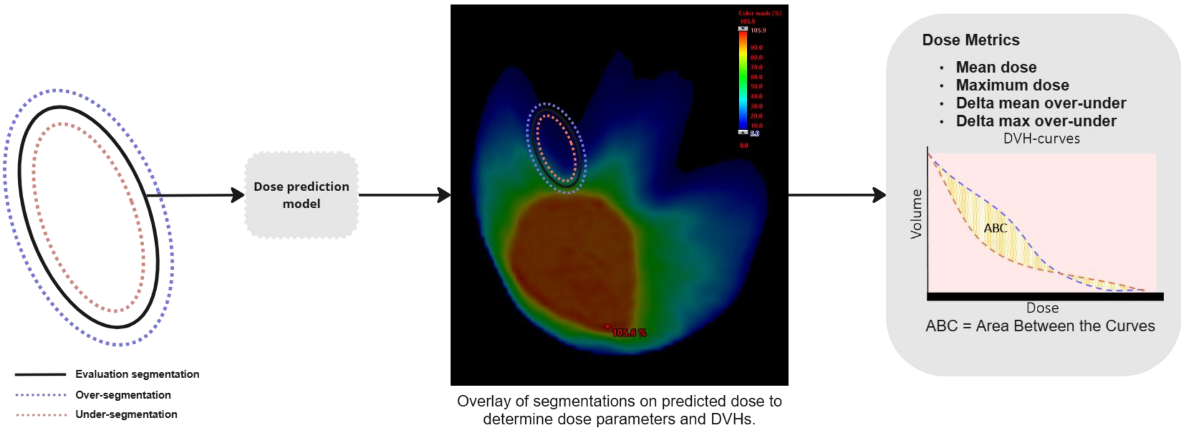

Figure 4: *Schematic overview showing how the dose and dose sensitivity are determined on the evaluation segmentation. Based on the segmentation result an over and under segmentation is determined. Both scenarios are used as input for the dose prediction model. Two different dose distributions will provide different dose parameters (mean dose and max dose) for the structure as well as different dose volume histogram (DVH) curves. The ABC, area between the curves as a fraction of the total DVH area is defined as a measure of the dosimetric impact.*

2.6 Evaluation of critical dose discriminator

Using inputs from both estimators, the evaluation assistant applies a decision scheme to flag contours requiring further review. Our analysis includes 17 organs across 30 cases (507 segmentations in total). Results are compared to reference data using a confusion matrix to determine true/false positives and negatives, enabling the calculation of sensitivity and specificity. Our goal is high sensitivity with acceptable specificity that eliminates a significant amount of non-critical cases. Selection criteria can be adjusted based on confusion matrix results Figure 5.

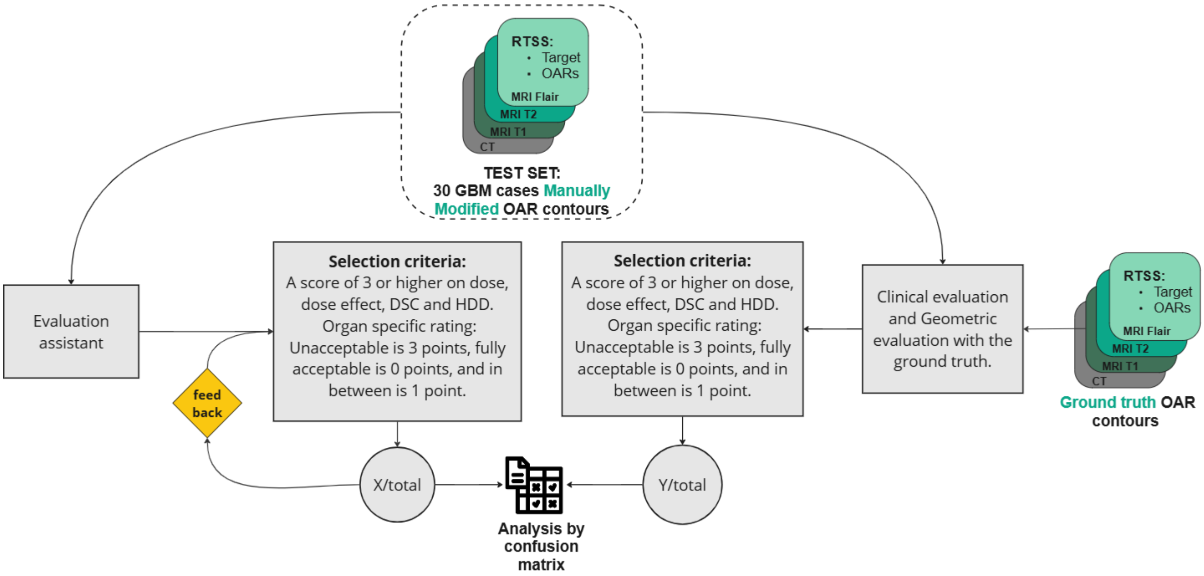

Figure 5: *Definition of the critical dosimetric events for both the clinical dosimetric evaluation used as the reference and for the critical dose estimator that we want to validate. A critical event is defined as either one or both conditions being met. Because the critical dose estimator requires a high sensitivity, additional safety*

margins are defined to reduce the chances of having false negatives. The critical events as a fraction of the total contours are compared for both methods in a confusion matrix.

2. Results traffic light vs random forest

Besides the evaluation assistant using the traffic light system, data driven machine learning approaches were tested to predict if a contour was geometrically and or dosimetrically critical to the treatment.

To do so we used the same cohort of patient and the same clinical validation data as the ground truth. We trained a standard random forest model that used as the input, the 4 metrics derived from the analysis of the auto-segmentation results, our independent segmentation model and the dose prediction model which were the, mean or maximum dose, the dose sensitivity, the DSC and the HD. As the ground truth we used the clinical validation data, with the binary metric, to flag, or not to flag, as the labels to train. With a total of 507 contours, we used a 4 fold cross validation where 75% of the data is used for training and 25 % (120 cases) is used for testing. For the best trained model we derived the confusion matrix, and calculated the sensitivity, specificity, positive predictive value and negative predictive value. The results are shown in figure 6, where they can be compared against the results of the evaluation assistant.

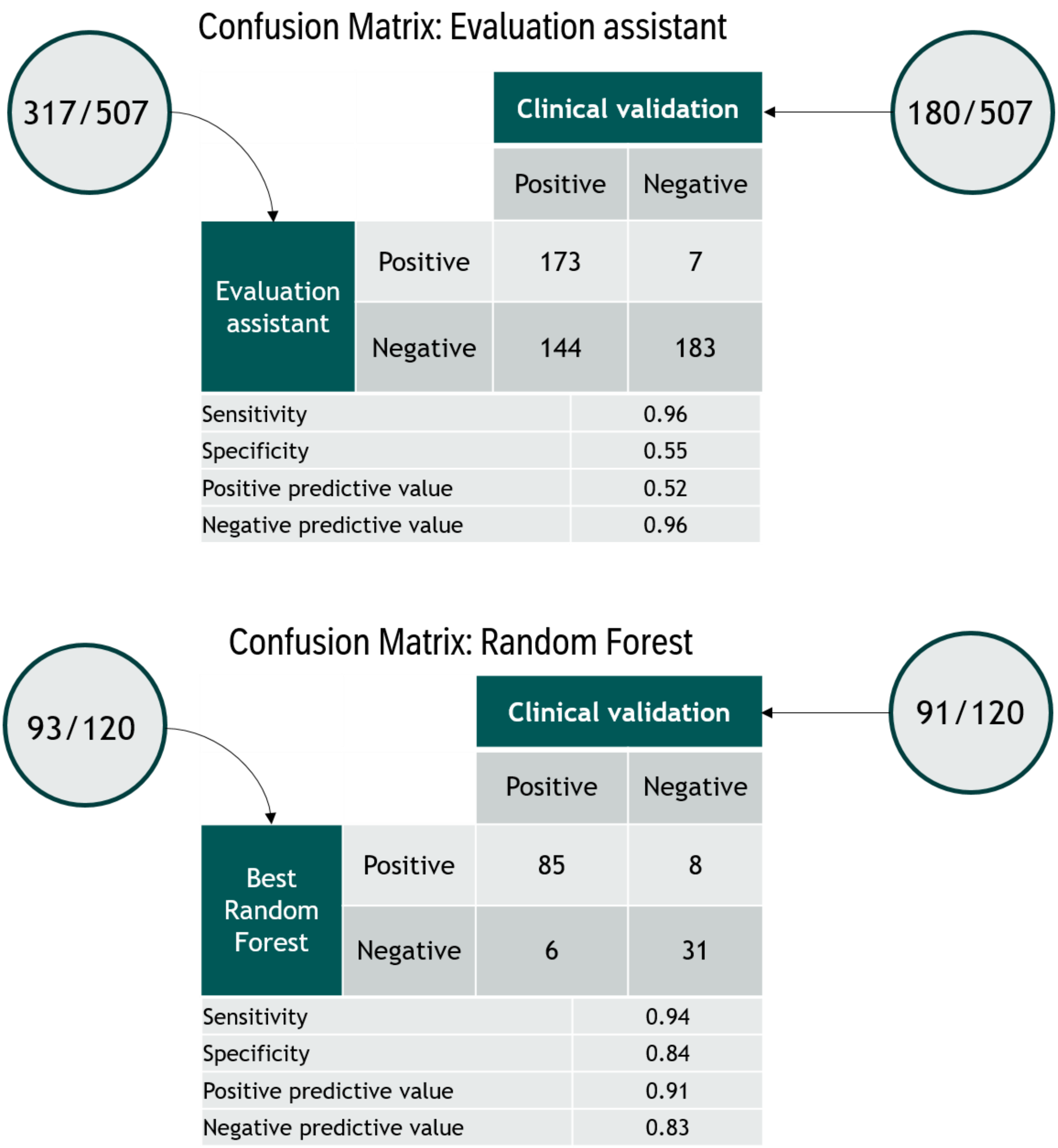

Figure 6: *On top, the confusion matrix comparing the results of the evaluation assistant to the results of the clinical evaluation. Below is the confusion matrix of the best performing random forest model. It should be noted that because part of the data was used during training the total amount of contours in the evaluation is only 120. The random forest provides a better specificity but this is at the expense of the sensitivity and the negative predictive value, which are critical for this type of quality assurance.*

3. References:

[1] Poel R, Kamath AJ, Willmann J, Andratschke N, Ermiş E, Aebersold DM, et al. Deep-Learning-Based Dose Predictor for Glioblastoma–Assessing the Sensitivity and Robustness for Dose Awareness in Contouring. Cancers (Basel) 2023;15. https://doi.org/10.3390/cancers15174226.

[2] Reason J. Human error: Models and management. Br Med J 2000;320:768–70. https://doi.org/10.1136/bmj.320.7237.768.

[3] Passarge M, Fix MK, Manser P, Stampanoni MFM, Siebers J V. A Swiss cheese error detection method for real-time EPID-based quality assurance and error prevention. Med Phys 2017;44:1212–23. https://doi.org/doi: 10.1002/mp.12142.

[4] Scoccianti S, Detti B, Gadda D, Greto D, Furfaro I, Meacci F, et al. Organs at risk in the brain and their dose-constraints in adults and in children: A radiation oncologist's guide for delineation in everyday practice. Radiother Oncol 2015;114:230–8. https://doi.org/10.1016/j.radonc.2015.01.016.

[5] Niyazi M, Brada M, Chalmers AJ, Combs SE, Erridge SC, Fiorentino A, et al. ESTRO-ACROP guideline "target delineation of glioblastomas." Radiother Oncol 2016;118:35–42. https://doi.org/10.1016/j.radonc.2015.12.003.

[6] Ronneberger O, Fischer P, Brox T. U-net: Convolutional networks for biomedical image segmentation. Lect Notes Comput Sci (Including Subser Lect Notes Artif Intell Lect Notes Bioinformatics) 2015;9351:234–41. https://doi.org/10.1007/978-3-319-24574-4_28.

[7] Abayazeed AH, Abbassy A, Müeller M, Hill M, Qayati M, Mohamed S, et al. NS-HGlio: A generalizable and repeatable HGG segmentation and volumetric measurement AI algorithm for the longitudinal MRI assessment to inform RANO in trials and clinics. Neuro-Oncology Adv 2023;5:1–10. https://doi.org/10.1093/noajnl/vdac184.

[8] Niyazi M, Andratschke N, Bendszus M, Chalmers AJ, Erridge SC, Galldiks N, et al. ESTRO-EANO guideline on target delineation and radiotherapy details for glioblastoma. Radiother Oncol 2023;184:109663. https://doi.org/10.1016/j.radonc.2023.109663.

[9] Van Esch A, Tillikainen L, Pyykkonen J, Tenhunen M, Helminen H, Siljamäki S, et al. Testing of the analytical anisotropic algorithm for photon dose calculation. Med Phys 2006;33:4130–48. https://doi.org/10.1118/1.2358333.

[10] Rüfenacht E, Kamath A, Suter Y, Poel R, Ermiş E, Scheib S, et al. PyRaDiSe: A Python package for DICOM-RT-based auto-segmentation pipeline construction and DICOM-RT data conversion. Comput Methods Programs Biomed 2023;231. https://doi.org/10.1016/j.cmpb.2023.107374.

[11] Liu S, Zhang J, Li T, Yan H, Liu J. Technical Note: A cascade 3D U-Net for dose prediction in radiotherapy. Med Phys 2021;48:5574–82. https://doi.org/10.1002/mp.15034.

[12] Babier A, Zhang B, Mahmood R, Moore KL, Purdie TG, McNiven AL, et al. OpenKBP: The open-access knowledge-based planning grand challenge and dataset. Med Phys 2021;48:5549–61. https://doi.org/10.1002/mp.14845.

[13] He K, Zhang X, Ren S, Sun J. Delving deep into rectifiers: Surpassing human-level performance on imagenet classification. Proc IEEE Int Conf Comput Vis 2015;2015 Inter:1026–34. https://doi.org/10.1109/ICCV.2015.123.

[14] Kamath A, Poel R, Willmann J, Andratschke N, Reyes M. How Sensitive Are Deep Learning Based Radiotherapy Dose Prediction Models To Variability In Organs At Risk Segmentation? Proc - Int Symp Biomed Imaging 2023;2023-April:1–4. https://doi.org/10.1109/ISBI53787.2023.10230559.

[15] Poel R, Kamath AJ, Willmann J, Andratschke N, Ermi E, Aebersold DM, et al. Deep-Learning-Based Dose Predictor for Glioblastoma – Assessing the Sensitivity and Robustness for Dose Awareness in Contouring. Cancers (Basel) 2023;15:1–13. https://doi.org/doi.org/10.3390/cancers15174226.

[16] Poel R, Rüfenacht E, Hermann E, Scheib S, Manser P, Aebersold DM, et al. The predictive value of segmentation metrics on dosimetry in organs at risk of the brain. Med Image Anal 2021;73:102161. https://doi.org/10.1016/j.media.2021.102161.

[17] Poel R, Rüfenacht E, Ermis E, Müller M, Fix MK, Aebersold DM, et al. Impact of random outliers in auto-segmented targets on radiotherapy treatment plans for glioblastoma. Radiat Oncol 2022;17:170. https://doi.org/10.1186/s13014-022-02137-9.
